# *AFG2B* gene variants and elevated protein expression in lupus nephritis: new insights into childhood-onset systemic lupus erythematosus

**DOI:** 10.1186/s42358-025-00509-9

**Published:** 2025-12-13

**Authors:** Paola Pinheiro Kahwage, Graziella Ribeiro de Sousa, Ronald Rodrigues de Moura, Fabiano Pinto Saggioro, Inalda Facincani, Denise do Nascimento Queiroga, Veridiana Kiill Suazo, Rosane Gomes de Paula Queiroz, Davi Casale Aragon, Virginia Paes Leme Ferriani, Paula Sandrin-Garcia, Luciana Martins de Carvalho

**Affiliations:** 1 Department of Pediatrics, Division of Pediatric Rheumatology, Ribeirão Preto Medical School, University of São Paulo, Ribeirão Preto, Brazil; 2 Department of Genetics, Ribeirão Preto Medical School, University of São Paulo, Ribeirão Preto, Brazil; 3 Department of Advanced Diagnostics, Institute for Maternal and Child Health- IRCCS “Burlo Garofolo”, Trieste, Italy; 4 Department of Medicine, Ribeirão Preto Medical School, University of São Paulo, Ribeirão Preto, Brazil; 5 Department of Pediatrics, Division of Pediatric Nephrology, Ribeirão Preto Medical School, University of São Paulo, Ribeirão Preto, Brazil; 6 Keizo Asami Institute / Genetics Department, Federal University of Pernambuco, Recife, Brazil; 7 Department of Pediatrics, Ribeirão Preto Medical School, University of São Paulo, Ribeirão Preto, Brazil; 8 Clinical Hospital of Ribeirão Preto Medical School, University of São Paulo – Brazil, Av. Bandeirantes, Ribeirão Preto, SP 3900, 14049-900 Brazil

**Keywords:** SPATA5L1, AFG2B, Lupus nephritis, Systemic lupus erythematosus, Childhood-onset

## Abstract

**Background:**

Previous genome-wide association studies (GWAS) have associated chronic kidney disease with *AFG2B* variants. The AFG2B protein is linked to key immune-regulating processes through its interaction with proteasome proteins implicated in systemic inflammation. Dysregulation of these processes is a key factor in the onset of childhood-onset systemic lupus erythematosus (cSLE). This study aimed to investigate the frequency of *AFG2B* gene variants and the renal expression of the AFG2B protein in cSLE.

**Methods:**

This two-phase, cross-sectional observational study included 45 patients with cSLE from a tertiary hospital in Brazil. In the first phase, we determined the frequency of variants in the *AFG2B* gene using PCR followed by Sanger sequencing. In the second phase, we analyzed AFG2B protein expression via immunohistochemistry in renal biopsies from patients with lupus nephritis (LN) and analyzed two publicly available databases from the Gene Expression Omnibus (GEO)—GSE32591 and GSE127797. To compare AFG2B expression in the LN, we used renal biopsy samples from patients undergoing nephrectomy for non-renal diseases (disease-free controls). We also performed modeling and molecular dynamics of AFG2B protein variants with pathogenic potential.

**Results:**

One patient with cSLE (2.2%) had a missense variant in exon 5 of the *AFG2B* gene (c.1774 A > G); molecular dynamics studies indicated this variant (p.N592D) is more stable than the wild-type protein. Twenty-five patients (55.5%) had an intronic variant (g.12211 A > G), and one patient (2.2%) had a novel intronic variant in heterozygosity (g.13196 A > G). Kidney tissue samples from patients with cSLE exhibited significantly increased AFG2B protein expression compared to normal kidney tissues (*p* < 0.01). We also observed a positive correlation between AFG2B protein expression in the tubules and the urinary protein-to-creatinine ratio (UPCR) in patients with cSLE.

**Conclusions:**

AFG2B protein expression is significantly elevated in the renal tissues of patients with LN compared to that in controls. Furthermore, its expression in renal tubules correlated positively with the UPCR. These results suggest a potential role for this gene in the pathophysiology of cSLE and warrant further functional investigation.

## Background

Previous genome-wide association studies (GWAS) have revealed a genetic correlation between *AFG2B* (AFG2 AAA ATPase Homolog B, formerly SPATA5L1) variants and chronic kidney disease [1, 2]. In line with these findings, we observed high AFG2B protein expression in the kidney of a 9-month-old patient diagnosed with full-house nephropathy associated with a missense variant in the *AFG2B* gene [3]. The AFG2B protein is involved in key processes regulating the immune system, including protein remodeling and degradation, ribosomal biogenesis and maturation, and the maintenance of genome integrity [4, 5]. It interacts with proteins that play a central role in proteasomal regulation and control of cell cycle progression and apoptosis, forming a complex network of protein-protein interactions. An imbalance of these mechanisms produces autoantibodies and triggers dysregulated inflammatory phenomena dominated by the secretion of type I interferons. This process suggests two patient phenotypes: one predominantly autoimmune and the other autoinflammatory [6]. Notably, interferon plays a role in both Mendelian and sporadic forms of systemic lupus erythematosus (SLE). However, research on monogenic childhood-onset SLE indicates that the disease’s pathways are distinct from those of the autoinflammatory interferonopathies [7].

Given its functional characteristics, the *AFG2B* gene has emerged as a promising candidate involved in the regulation of immune tolerance mechanisms. The breakdown of immune tolerance is a key event in the pathogenesis of childhood-onset systemic lupus erythematosus (cSLE). Therefore, we hypothesized that AFG2B contributes to the modulation of the inflammatory response in lupus nephritis (LN), as evidenced by its increased expression in the tubular and glomerular compartments of affected patients. Furthermore, we postulate that enhanced renal AFG2B protein expression is associated with disease severity, as indicated by clinical parameters such as elevated proteinuria levels.

This study aimed to determine the frequency of *AFG2B* gene variants in cSLE and assess renal AFG2B protein expression.

## Methods

We conducted a cross-sectional observational study of 45 cSLE patients recruited via convenience sampling from the Pediatric Rheumatology Outpatient clinic of the Clinical Hospital of the Faculty of Medicine of Ribeirão Preto, University of São Paulo, between January 2019 and February 2020. All patients fulfilled the 2012 Systemic Lupus International Collaborating Clinics (SLICC) classification criteria for SLE [8], with disease onset before 18 years of age.

The study was conducted in two phases. In the first phase, we determined the frequency of variants in the *AFG2B* gene. In the second, we analyzed AFG2B protein expression via immunohistochemistry in renal biopsies from patients with LN, which were performed as part of their routine clinical care. Renal biopsy samples from post-accident nephrectomy patients were obtained from the hospital’s pathology and forensic medicine services to serve as non-inflammatory controls. Due to the limited availability of pediatric disease-free renal biopsy samples, adult controls were predominantly used. Although there are known structural and functional differences between adult and pediatric renal tissue—such as glomerular size, nephron maturity, and filtration capacity—this approach was justified by the similarity in pathological patterns and the shared classification criteria of LN in both age groups. Additionally, the inclusion of a 14-year-old control subject exhibiting AFG2B protein expression profiles comparable to adult controls, along with a median of cSLE cohort age of 11 years, supports the reliability of the results.

### *AFG2B* gene sequencing

We collected approximately 10 mL of blood, with 5 mL in an EDTA anticoagulant tube and 5 mL in a dry tube. DNA was then isolated using DNAzol according to the manufacturer’s protocol. To sequence the *AFG2B* gene, we performed PCR using the primers detailed in Table 1. Primers were designed to target each of the eight exons and approximately 100 flanking base pairs, yielding an expected product size of 300–500 base pairs (bp). Furthermore, these target regions were selected for low GC content (≤ 50%) to ensure a moderate annealing temperature (55–65 °C), as suggested by the Tm Calculator tool (https://www.thermofisher.com/br/en/home/brands/thermo-scientific/molecular-biology/molecular-biology-learning-center/molecular-biology-resource-library/thermo-scientific-web-tools/tm-calculator.html). Primer quality was verified using the OligoAnalyzer tool (https://www.idtdna.com/pages/tools/oligoanalyzer, RRID: SCR_001363), and primer specificity was confirmed using the UCSC In-Silico PCR tool (https://genome.ucsc.edu/cgi-bin/hgPcr, RRID: SCR_003089).

Following amplification, the PCR products (240–450 bp) were visualized on a 2% agarose gel to confirm fragment specificity and size. The products were then purified with Exo-SAP-IT (USB Corporation, Cleveland, OH) and directly sequenced using the BigDye Terminator v1.1 Cycle Sequencing Kit (Applied Biosystems, Foster City, CA).

Finally, we performed the bioinformatics analysis. The resulting sequences were analyzed with Codon Code Aligner software to identify variants. We assessed allele frequency using data from gnomAD v4.1.0 (https://gnomad.broadinstitute.org) and predicted variant pathogenicity using the CADD, REVEL, and SpliceAI (delta) score.

**Table 1:** Primers used for sequencing the exons of the *AFG2B* gene

Exon	Gene	Primers	Fragment size
1	AFG2B 1 F AFG2B 1R	GTGCTCCTGGCGGGGCC TGCTCGACTGCCCCGCTG	240 pb
2	AFG2B 2 F AFG2B 2R	TGTACCCATGAGCCAACC TAAGGAAAATGTACTCACCGC	444 pb
3	AFG2B 3 F AFG2B 3R	GGCAGGGAGACATTTAATTCTT AGAGCAGTTTCAGCGCAGTATG	416 pb
4	AFG2B 4 F AFG2B 4R	GGAGGCTGAGAATTGTTAAA TAACTGGAAAAAACACTAGTAA	409 pb
5	AFG2B 5 F AFG2B 5R	GAGCAACAGAGCAAGACTGTC TACCTCACCTTTCCTAGCC	514 pb
6	AFG2B 6 F AFG2B 6R	ACACAAAGTAGCTCTTTGGT CCATGGACACCTATATCCAAG	404 pb
7	AFG2B 7 F AFG2B 7R	CTCGGTAATGTGAAAGGAAACT CATGGTACCACACAGGCTT	332 pb
8	AFG2B 8 F AFG2B 8R	GCCTGGGCAATAAGAGCAA CGACAGGCAAGTTCACATT	312 pb

### Immunohistochemistry

AFG2B protein expression was evaluated via immunohistochemistry. Staining was performed using a primary anti-AFG2B antibody (1:50 dilution) (ab126354, RRID: AB_11128554, Abcam, Cambridge, United Kingdom) applied overnight. The signal was then detected using the EnVision™ polymer detection system (Dako, Glostrup, Denmark).

To semi-quantify immunostaining intensity, tubules and glomeruli were analyzed separately in immunohistochemistry images using ImageJ software (version 2.0, RRID: SCR_002285), following the method described by Patera et al. (2019) [9]. Representative images of tubules and glomeruli were converted into three independent digital images (H&E, DAB, and a complementary image). The resulting DAB images were converted to 8-bit grayscale, and two representative fields containing manually selected tubules and glomeruli of interest were chosen for analysis. From each field, the mean gray value was measured. Optical density (OD) was then calculated using the standard formula: OD = log (maximum intensity / mean intensity), where the maximum intensity for an 8-bit image is 255. All analysis was performed by a single observer who was blinded to the experimental conditions.

### In silico analysis

**Fig. 1:**
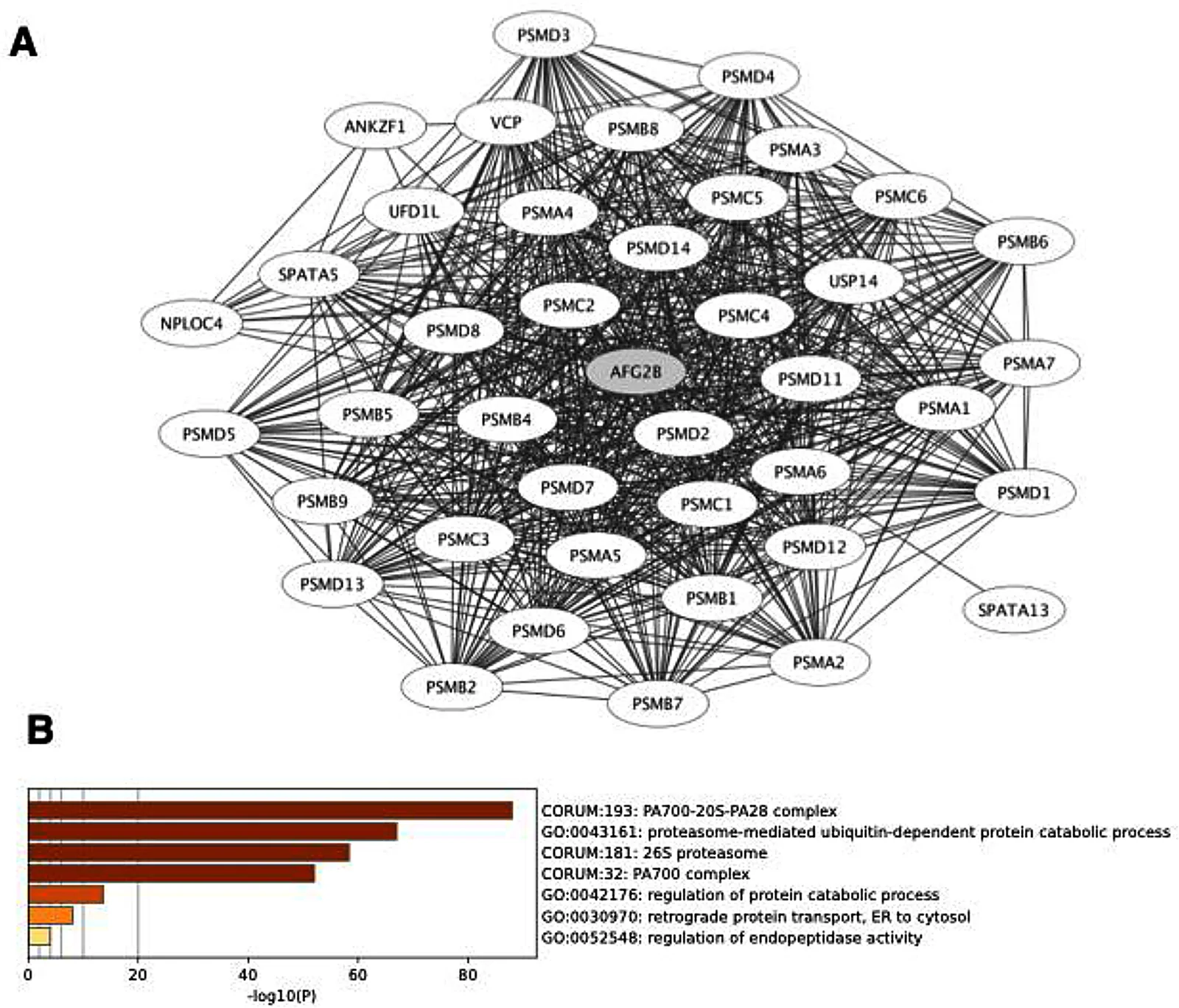
The protein-protein interaction (PPI) network of AFG2B and associated protein enrichment analysis. (**a**) The PPI network was generated using the STRING database [11]. The nodes and interactions were visualized in the Cytoscape tool [12]. (**b**) Metascape was used to analyze the enrichment of proteins within the AFG2B-regulated biomolecular network, which identified seven distinct clusters [13]

To explore the interactions of AFG2B with other proteins and elucidate potential biological processes within its network, we constructed a protein-protein interaction (PPI) network using the G database (version 12.0, RRID: SCR_005223) and visualized the nodes with software (version 3.10.3, RRID: SCR_003032). Functional enrichment analysis was then performed using the Metascape database (Fig. 1).

To investigate the structural impact of the N592D and S273C mutations on AFG2B, we performed a 100 ns molecular dynamics (MD) simulation. The AFG2B protein sequence was retrieved from the Uniprot Knowledge Base (UniprotKB) using the access identifier Q9BVQ7. The wild-type structure was subsequently modeled using the I-TASSER (RRID: SCR_014627) web server with default parameters and refined using 3DRefine software (RRID: SCR_021883). The quality of the refined model was evaluated using the MolProbity score (RRID: SCR_014226) and validated with ERRAT (RRID: SCR_001921), PROCHECK (RRID: SCR_019043), and ProSAS (RRID: SCR_007876) software, all with default parameters. Validation confirmed that the modeled structure was adequate for MD simulations (PROCHECK: PASS; ERRAT score: 88.5%; ProSAS: PASS). Mutations were introduced into the wild-type structure using the Mutagenesis tool in PyMOL (https://pymol.org/2/, RRID: SCR_000305).

Molecular dynamics simulations of the wild-type and mutated protein structures were conducted using GROMACS 2020 (RRID: SCR_014565) [10]. Each complex was placed in a 0.7 nm cubic box and solvated with SPC-E water molecules. Sodium and chlorine ions were added to neutralize the system. Following energy minimization, the system was equilibrated for 10 ns (temperature and pressure), and the production phase was run for 100 ns. To assess structural stability, we calculated the root-mean-squared deviation (RMSD), root-mean-squared fluctuation (RMSF), radius of gyration (Rg), and solvent-accessible surface area.

### *AFG2B* gene expression in renal biopsy

To evaluate *AFG2B* gene expression in human (LN) samples, we analyzed two publicly available Gene Expression Omnibus (GEO) datasets (GSE32591 [14] and GSE127797 [15]) using the GEO2R web tool (RRID: SCR_016569). Expression data in both datasets were generated using the Affymetrix Human Transcriptome Array 2.0.

The GSE32591 dataset [14] contains 47 renal biopsies from 32 patients with LN and 15 healthy controls. The GSE127797 dataset [15] contains renal biopsies from 88 patients with LN, microdissected into 47 tubulointerstitial and 41 glomerular samples. For both datasets, data were obtained as log2-transformed normalized expression values. The samples were categorized by their microdissected component (tubules, interstitium, or glomerulus) for comparative transcriptome analysis.

### Statistical analysis

Multiple linear regression models were used to assess the association between tubular and glomerular AFG2B expression and several clinical and pathological variables. These categorical variables included: creatinine clearance (average vs. altered), urinary protein-to-creatinine ratio (UPCR; nephrotic vs. non-nephrotic), activity index (< 12 vs. ≥12), renal biopsy chronicity index (yes vs. no), gender, BILAG score [16] (A, B, or C), and ISN/RPS histological class [17] (I–V). The models were adjusted for disease duration, sex, age, and immunosuppressive therapy as covariates. A false discovery rate (FDR) correction was applied to all models.

Spearman correlation analysis was performed to evaluate the relationship between tubular and glomerular AFG2B expression and several continuous variables: erythrocyte sedimentation rate (ESR), C-reactive protein (CRP), SLEDAI-2k [18], and UPCR.

For group comparisons, Student’s t-test was used for two-group analyses (GSE127797), while one-way ANOVA with Bonferroni multiple comparison post-hoc test was used for analyses involving more than two groups (GSE32591). Log2 fold change (Log2FC) values were calculated as the mean difference between groups, and 95% confidence intervals (CIs) were reported.

All statistical analyses were performed using R Project for Statistical Computing (version 4.1.1, RRID: SCR_001905) and SAS (version 9.4, RRID: SCR_008567). A p-value < 0.05 was considered statistically significant.

## Results

This study included 45 patients with cSLE, whose clinical data are summarized in Table 2. Twenty-nine LN biopsies were performed during the follow-up period until data were collected. Based on the ISN/RPS classification, 51.7%, 13.7%, 10.3%, 6.2%, and 3.4% of biopsies were classified as classes IV, II, class IV + V, I, and I + III, respectively. Most patients (91.6%) had moderate-to-high SLEDAI-2 K disease activity index scores (≥ 5).

**Table 2:** Demographic, clinical, and laboratory characteristics at diagnosis of 45 patients with childhood-onset systemic lupus erythematosus

Characteristics	*n* (%)
**Demographic**	
Female gender	35 (77.7%)
Whites	29 (64.4%)
Diagnosis before puberty	19 (42.2%)
Median age (range)	11 (6–16 years old)
**Clinical**	
Renal abnormalities	35 (77.7)
Chronic kidney disease	3 (6.6)
Malar rash	23 (51.1)
Discoid lupus	3 (6.6)
Photosensitivity	23 (51.1)
Oral ulcers	9 (20%)
Non-scarring alopecia	12 (26.6)
Arthritis	28 (62.6)
Serositis	12 (26.6)
Neurological abnormalities	13 (28.8)
**Laboratory**	
Hematological abnormalities	30 (66.6)
Autoimmune hemolytic anemia	24 (53.3)
Leukopenia < 4000/mm^3^ or lymphopenia < 1000/mm^3^	13 (28.8)
Thrombocytopenia < 100 000/mm^3^	12 (26.6)
Complement consumption (C3/C4)	36 (80)
Anti-DNA	35 (77.7)
Anti-Sm (*n* = 42)	23 (54.7)
Antinuclear factor (ANA)	45 (100)
Antiphospholipid	23 (51.1)
Anticardiolipin (*n* = 44)	19 (43.1)
Anti beta2glycoprotein (*n* = 39)	8 (20.5)
Renal biopsy	29 (64.4)

As controls, we included four renal biopsies obtained from four controls (all males; 50% white; age, 14–58 years).

### *AFG2B* gene variants

Among the 45 cSLE patients evaluated, one (2.2%) had a rare heterozygous variant (c.1774 A > G; rs16943025) in exon 5 of the *AFG2B* gene. Based on current evidence and ACMG guidelines, this variant is best classified as a Variant of Uncertain Significance (VUS). It is predicted to be potentially damaging by in silico tools (CADD = 26.3, REVEL = 0.50) and has a general population allele frequency of 0.0019 (gnomAD database). This variant results in a substitution of asparagine with aspartic acid at position 592 of the protein (p.Asn592Asp).

**Fig. 2:**
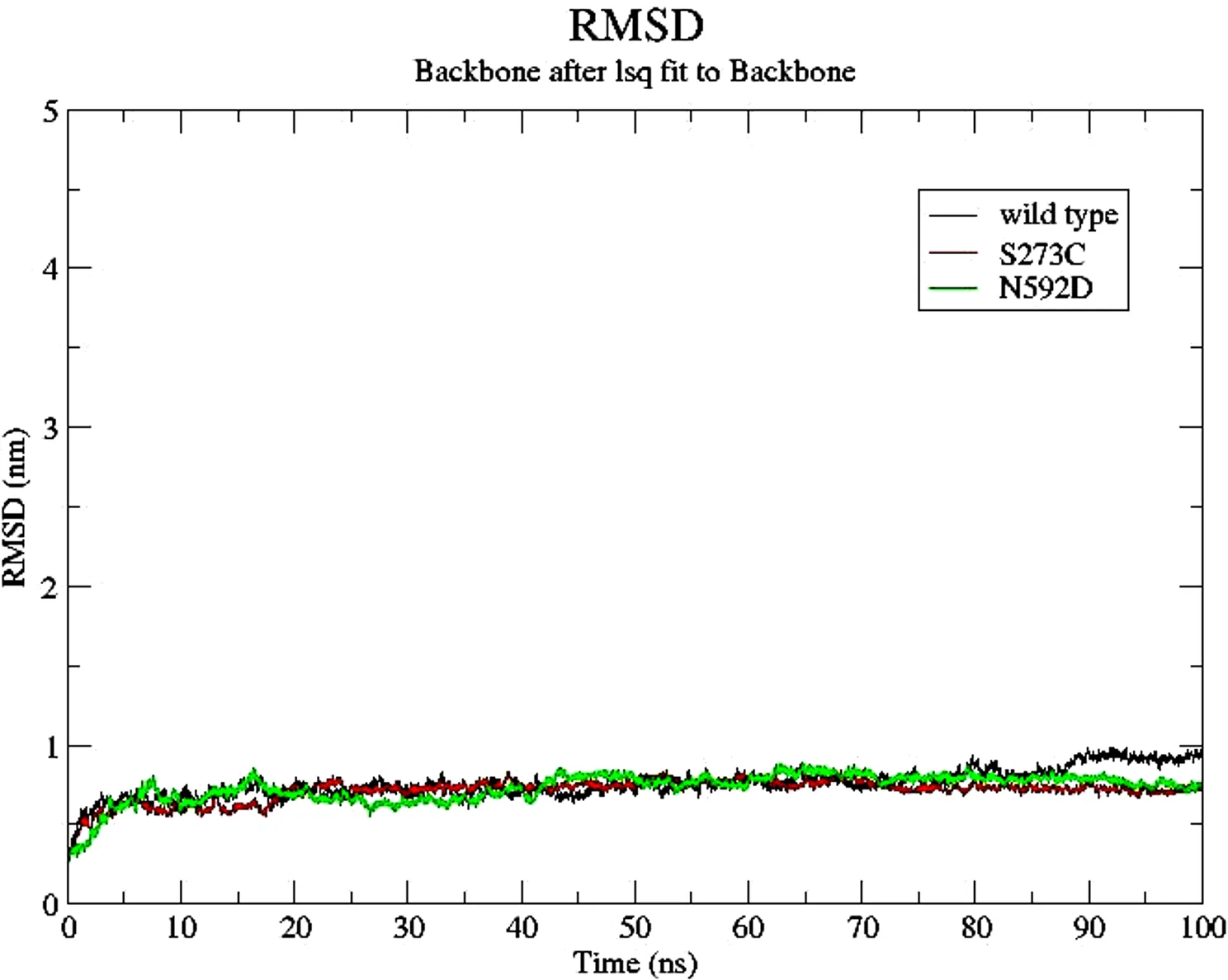
Root mean squared deviation (RMSD) values during molecular dynamics simulation for three *AFG2B* variants: the wild-type protein; the N592D mutant (observed in this study); and the S273C mutant (previously reported [3]). Molecular modeling was performed using the I-TASSER web server with default parameters and subsequently refined with the 3Drefine software

Validation checks confirmed the modeled structure’s suitability for MD simulations (PROCHECK: PASS; ERRAT: 88.5%; ProSA: PASS). The MD analysis indicated that the p.Arg592Asp (N592D) variant may impact the protein’s overall stability and compactness. After 75 ns, the wild-type protein structure began to lose stability, whereas the N592D variant remained stable until the simulation ended. This finding was supported by differences in the Solvent Accessible Surface Area (SASA) plot values (Fig. 2), which showed the N592D variant was more compact (i.e., had a lower solvent-accessible area) than the wild-type protein. The radius of gyration and RMSF analyses did not indicate any significant changes.

In addition to this pathogenic variant, two intronic variants were identified. First, a common intronic variant (g.12211 A > G; rs12440038) was identified in 25 cSLE patients (55.5%), 19 of whom (76%) were heterozygous. Given its high frequency in the general population (~ 45%), this variant is likely benign and its role in cSLE, if any, requires further investigation. Second, one patient (2.2%) harbored a novel, heterozygous intronic variant (g.13196 A > G) near exon 4. This variant, unreported in public databases (gnomAD, ABraOM, dbSNP),, also fits the VUS classification, and SpliceAI prediction yielded a delta score of 0.01–0.02, suggesting a low probability of impacting splicing [19].

### Evaluation of AFG2B protein and gene expression in renal biopsies

**Fig. 3:**
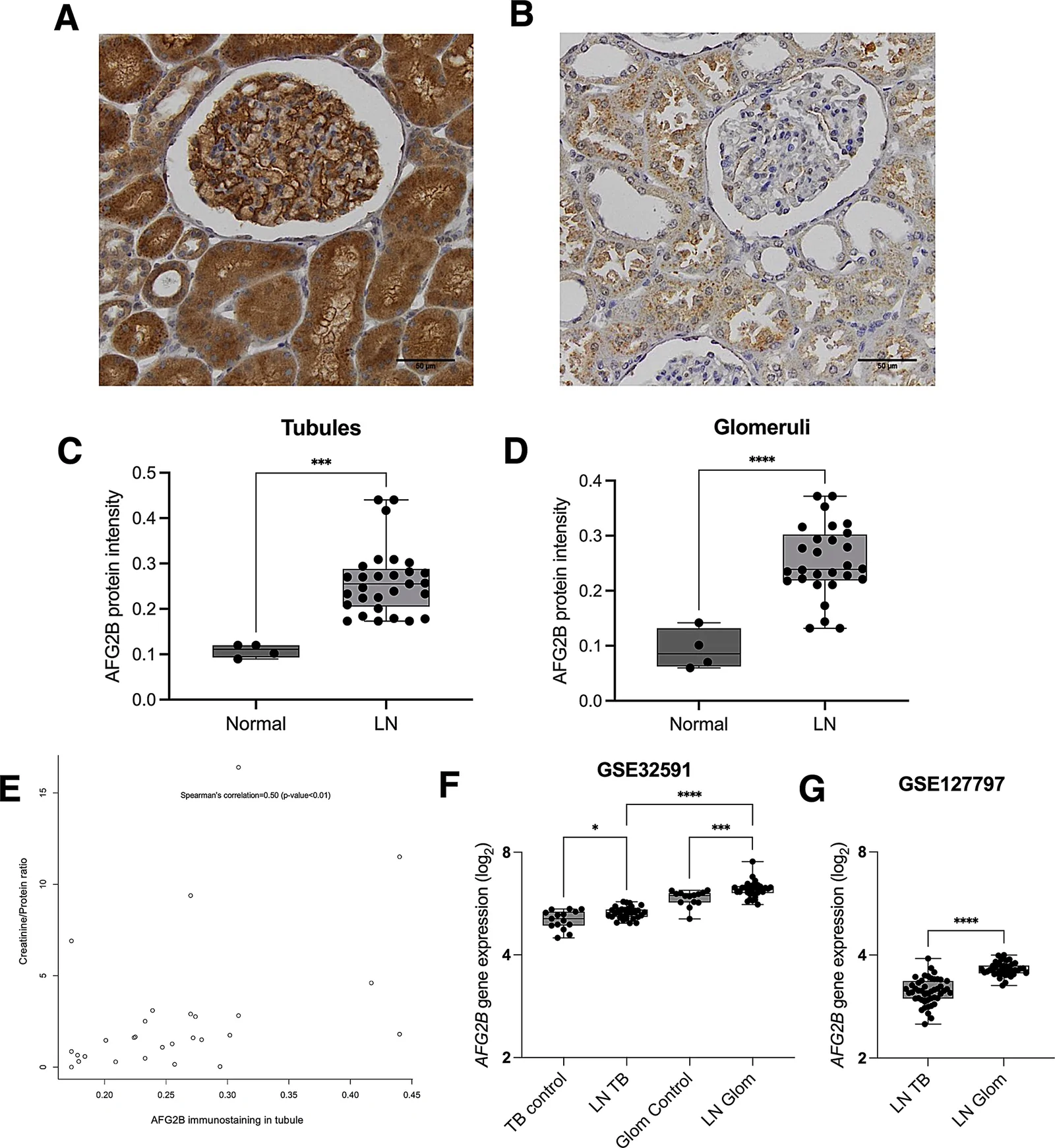
Evaluation of AFG2B protein. (**a**, **b**) Representative images of AFG2B immunostaining in renal biopsies from patients with LN (**a**) and normal controls (**b**). Original magnification, 200x. (**c**, **d**) Semi-quantification of AFG2B immunostaining in tubules (**c**) and glomeruli (**d**). (**e**) Spearman correlation between AFG2B protein expression in tubules and the urinary protein-to-creatinine ratio (UPCR). (**f**, **g**) AFG2B gene expression from public datasets GSE32591 (**f**) and GSE127797 (**g**). Statistical analyses were performed using Student’s t-test (**c**, **d**, **g**) and one-way ANOVA with Bonferroni multiple comparisons post-hoc test (f). *p ≤ 0.05, ***p ≤ 0.001, ****p ≤ 0.0001. TB = tubule and Glom = glomeruli

Immunohistochemistry assays of the LN renal biopsy samples revealed intense immunostaining for the AFG2B protein in the tubules and glomeruli (Fig. 3a). Compared with normal kidney controls, AFG2B protein expression was significantly higher (Figs. 3a-d, *p* < 0.001).

Analysis of AFG2B protein expression in renal biopsy samples from LN patients, alongside laboratory and histological parameters, revealed a moderate positive correlation between AFG2B expression in tubules and the UPCR (Spearman correlation coefficient = 0.5; *p* < 0.01) (Fig. 3e). However, AFG2B expression in glomeruli did not correlate with the UPCR. Furthermore, we found no associations between the intensity of AFG2B protein expression and acute phase proteins, SLEDAI and BILAG indices, creatinine clearance, LN histological class, proteinuria levels (nephrotic or non-nephrotic), activity index, or renal biopsy chronicity index, even in adjusted models.

Moreover, analysis of publicly available datasets showed significantly higher *AFG2B* gene expression in LN samples than in normal renal biopsy samples, both in tubules (log2FC: 0.23, CI: 0.016–0.46) and glomeruli (log2FC: 0.35, CI 95%: 0.12–0.58) (Fig. 3f). This elevated expression in glomeruli was particularly evident in datasets GSE32591 (log2FC: 0.90, CI 95%: 0.73–1.08) and GSE127797 (log2FC: 0.50, CI 95%: 0.40–0.60) (Figs. 3f–g). These findings are consistent across independent datasets, supporting the reproducibility of AFG2B upregulation in LN renal tissue. The magnitude of the logFC and narrow confidence intervals indicate a robust increase, corroborating our protein-level findings.

## Discussion

We found a 60% frequency of *AFG2B* gene variants among the patients: 25 patients had intronic variants and one patient had a missense variant. The g.12,211 A > G gene variant was identified in 55% of the patients. As this is a common polymorphism also observed in ~ 45% of the general population, it is unlikely to be a direct monogenic cause of cSLE and is probably a benign variant. Intronic variants located in regulatory sites involved in alternative splicing, such as intron retention, are known to alter messenger RNA and protein expression [20]. Therefore, despite its intronic location, this high-frequency variant warrants further investigation. Future studies will recruit patients from other centers dedicated to cSLE and include a matched control group.

The patient carrying the missense *AFG2B* gene variant (c.1774 A > G), which has a 1% frequency in the general population (Ensembl.org), developed cSLE before puberty and presented with class IV LN as the initial manifestation. While our MD studies suggested this variant could alter protein stability, this finding is exploratory, and the functional consequences of this increased stability for immune regulation and LN pathogenesis remain unknown. Therefore, a causative role for this specific VUS in the patient’s disease cannot be established.

Few studies have associated specific phenotypes with the *AFG2B* gene. To date, GWAS have linked SNPs in the *AFG2B* gene to CKD and glomerular filtration rate, while case reports have associated variants in the *AFG2B* and *AFG2A* genes with phenotypes such as hearing loss [21], cerebral palsy, and epilepsy [22, 23]. Previously, we associated a missense variant of the *AFG2B* gene with full-house nephropathy [3].

Despite the limited research on the *AFG2B* gene, the potential interactions of its protein with proteins of the proteasome complex and with those involved in cell cycle and apoptosis control make this gene a promising candidate in the regulation of immune diseases, including cSLE. Autoantigens, which are targets of systemic autoimmune diseases such as SLE, are often associated with active cellular processes, including cell division, proliferation, and transcription. Furthermore, nuclear proteasomes process self-antigens and alter antigen processing by generating high levels of antigenic peptides, changing the antigenic structure, or both. Therefore, previously cryptic epitopes are exposed and misrecognized as non-self by T cells, initiating a systemic autoimmune response [24].

Diseases caused by loss-of-function mutations in components of the 20 S proteasome—including PSMA3, PSMB8, PSMB9, PSMB4, and PSMB1—are associated with proteostasis defects and interferon production. These diseases are called proteasome-associated autoinflammatory syndromes (PRAAS) and present with varied clinical manifestations, such as periodic fevers, skin rashes, hepatosplenomegaly, and elevated acute phase reactants [7]. The direct interaction of *AFG2B* with proteins central to proteasomal regulation suggests its potential involvement in the induction or pathogenesis of cSLE. Our study supports this hypothesis by demonstrating increased AFG2B protein expression in the tubules and glomeruli of LN patients compared to controls.

To date, AFG2B protein expression in LN renal biopsy samples has not been described. However, publicly available GEO database records for human LN renal biopsy samples also indicate higher levels of *AFG2B* gene expression, suggesting that the *AFG2B* gene may plays a role in LN pathogenesis.

We found that tubular AFG2B protein expression in patients with LN was positively associated with the UPCR, whereas glomerular expression was not.

Given that previous studies have associated AFG2B SNPs with CKD [1, 2], the positive association between tubular AFG2B protein expression and the UPCR may reflect AFG2B protein signaling within the intense inflammatory and reparative cascade in this renal area. One hypothesis is that high concentrations of albumin may positively regulate pro-inflammatory, fibrogenic, and apoptotic genes, including AFG2B, in proximal tubule cells.

This study has some limitations. First, the sample size was small. In addition, the limited availability of disease-free renal biopsy samples from pediatric controls necessitated the use of adult renal biopsies as the primary comparison group. Finally, the absence of a control group from the same population precluded a more robust assessment of AFG2B polymorphisms. Despite these limitations, this study is the first to explore the renal expression of the *AFG2B* gene in cSLE patients, and its findings suggest a potential role for AFG2B in the disease’s pathophysiology, highlighting the protein as a promising target for further investigation.

## Conclusions

A high frequency (60%) of *AFG2B* gene variants, primarily intronic, was observed in patients with cSLE. AFG2B protein expression was also significantly increased in glomeruli and tubules from lupus nephritis samples compared to controls. Furthermore, this expression in renal tubules correlated positively with the UPCR. These results suggest a potential role for AFG2B in the pathophysiology of cSLE, thereby warranting further functional investigation.

## Data Availability

The Gene Expression Omnibus (GEO) datasets analyzed during the current study are publicly available in the GEO repository, under accession numbers GSE32591 [14] and GSE127797 [15]. The remaining datasets generated and/or analyzed during the current study are available from the corresponding author on reasonable request.

## Abbreviations

AFG2B: ATPase family gene 2 B
Anti-DNA: Anti-deoxyribonucleic acid
Anti-Sm: Anti-Smith
BILAG: British Isles Lupus Assessment Group
CRP: C-reactive protein
Csle: Childhood-onset systemic lupus erythematosus
ESR: Erythrocyte sedimentation rate
GWAS: Genome-wide association studies
ISN/RPS: International Society of Nephrology/Renal Pathology Society
LN: Lupus nephritis
SLEDAI- 2K: Systemic Lupus Erythematosus Disease Activity Index 2000
SNPs: Single-stranded polymorphisms
SPATA5L1: Spermatogenesis Associated 5 Like 1

## References

[1] Kubo Y, Imaizumi T, Ando M, Nakatochi M, Yasuda Y, Honda H, et al. Association between kidney function and genetic polymorphisms in atherosclerotic and chronic kidney diseases: A cross-sectional study in Japanese male workers. PLoS ONE. 2017;12:e0185476. 29016630 10.1371/journal.pone.0185476 PMC5634546

[2] Köttgen A, Glazer NL, Dehghan A, Hwang SJ, Katz R, Li M, et al. Multiple loci associated with indices of renal function and chronic kidney disease. Nat Genet. 2009;41:712–7. 19430482 10.1038/ng.377 PMC3039280

[3] de Carvalho LM, de Sousa GR, Moura R, Saggioro F, Facincani I, Costa R, et al. Full-house nephropathy associated with high expression of SPATA5L1 due to a genetic pathogenic variant. Rheumatol (Oxf Engl). 2022;61:e84–6. 10.1093/rheumatology/keab896 34864870

[4] Krishnamoorthy V, Foglizzo M, Dilley RL, Wu A, Datta A, Dutta P, et al. The SPATA5-SPATA5L1 ATPase complex directs replisome proteostasis to ensure genome integrity. Cell. 2024;187:2250–e6831. 38554706 10.1016/j.cell.2024.03.002 PMC11055677

[5] Ni C, Schmitz DA, Lee J, Pawłowski K, Wu J, Buszczak M. Labeling of heterochronic ribosomes reveals C1ORF109 and SPATA5 control a late step in human ribosome assembly. Cell Rep. 2022;38:110597. 35354024 10.1016/j.celrep.2022.110597 PMC9004343

[6] Tesser A, de Carvalho LM, Sandrin-Garcia P, Pin A, Pastore S, Taddio A, et al. Higher interferon score and normal complement levels May identify a distinct clinical subset in children with systemic lupus erythematosus. Arthritis Res Ther. 2020;22:91. 32334613 10.1186/s13075-020-02161-8 PMC7183668

[7] Goldbach-Mansky R, Alehashemi S, de Jesus AA. Emerging concepts and treatments in autoinflammatory interferonopathies and Monogenic systemic lupus erythematosus. Nat Rev Rheumatol. 2025;21:22–45. 39623155 10.1038/s41584-024-01184-8

[8] Petri M, Orbai AM, Alarcón GS, Gordon C, Merrill JT, Fortin PR, et al. Derivation and validation of the systemic lupus international collaborating clinics classification criteria for systemic lupus erythematosus. Arthritis Rheum. 2012;64:2677–86. 22553077 10.1002/art.34473 PMC3409311

[9] Patera F, Cudzich-Madry A, Huang Z, Fragiadaki M. Renal expression of JAK2 is high in polycystic kidney disease and its Inhibition reduces cystogenesis. Sci Rep. 2019;9:4491. 30872773 10.1038/s41598-019-41106-3 PMC6418191

[10] Alarcón-Riquelme ME. The genetics of systemic lupus erythematosus: Understanding how SNPs confer disease susceptibility. Springer Semin Immunopathol. 2006;28:109–17. 16964481 10.1007/s00281-006-0033-4

[11] von Mering C, Huynen M, Jaeggi D, Schmidt S, Bork P, Snel B. String: a database of predicted functional associations between proteins. Nucleic Acids Res. 2003;31:258–61. 12519996 10.1093/nar/gkg034 PMC165481

[12] Shannon P, Markiel A, Ozier O, Baliga NS, Wang JT, Ramage D, et al. Cytoscape: a software environment for integrated models of biomolecular interaction networks. Genome Res. 2003;13:2498–504. 14597658 10.1101/gr.1239303 PMC403769

[13] Zhou Y, Zhou B, Pache L, Chang M, Khodabakhshi AH, Tanaseichuk O, et al. Metascape provides a biologist-oriented resource for the analysis of systems-level datasets. Nat Commun. 2019;10:1523. 30944313 10.1038/s41467-019-09234-6 PMC6447622

[14] Berthier CC, Bethunaickan R, Gonzalez-Rivera T, Nair V, Ramanujam M, Zhang W et al. Cross-species transcriptional network analysis defines shared inflammatory responses in murine and human lupus nephritis. J Immunol. 10.4049/jimmunol.1103031 PMC3392438 22723521

[15] Almaani S, Prokopec SD, Zhang J, Yu L, Avila-Casado C, Wither J, et al. Rethinking lupus nephritis classification on a molecular level. J Clin Med. 2019;8:1524. 31547518 10.3390/jcm8101524 PMC6832959

[16] Isenberg DA, Rahman A, Allen E, Farewell V, Akil M, Bruce IN, et al. BILAG 2004. Development and initial validation of an updated version of the British Isles lupus assessment group’s disease activity index for patients with systemic lupus erythematosus. Rheumatol (Oxf Engl). 2005;44:902–6. 10.1093/rheumatology/keh624 15814577

[17] Bajema IM, Wilhelmus S, Alpers CE, Bruijn JA, Colvin RB, Cook HT, et al. Revision of the international society of Nephrology/Renal pathology society classification for lupus nephritis: clarification of definitions, and modified National institutes of health activity and chronicity indices. Kidney Int. 2018;93:789–96. 29459092 10.1016/j.kint.2017.11.023

[18] Gladman DD, Ibañez D, Urowitz MB. Systemic lupus erythematosus disease activity index 2000. J Rheumatol. 2002;29:288–91. 11838846

[19] Jaganathan K, Kyriazopoulou Panagiotopoulou SK, McRae JF, Darbandi SF, Knowles D, Li YI, et al. Predicting splicing from primary sequence with deep learning. Cell. 2019;176:535–e4824. 30661751 10.1016/j.cell.2018.12.015

[20] Zheng JT, Lin CX, Fang ZY, Li HD. Intron retention as a mode for RNA-Seq data analysis. Front Genet. 2020;11:586. 32733531 10.3389/fgene.2020.00586 PMC7358572

[21] Braun F, Hentschel A, Sickmann A, Marteau T, Hertel S, Förster F et al. Muscular and molecular pathology associated with SPATA5 deficiency in a child with EHLMRS. Int J Mol Sci. 2021;22. 10.3390/ijms22157835 PMC8345956 34360601

[22] Oesch G, Bozarth XL. Rufinamide efficacy and association with phenotype and genotype in children with intractable epilepsy: A retrospective single center study. Epilepsy Res. 2020;168:106211. 31575436 10.1016/j.eplepsyres.2019.106211

[23] Zanus C, Costa P, Faletra F, Musante L, Russo A, Grazian L, et al. Description of a peculiar alternating ictal electroclinical pattern in a young Boy with a novel SPATA5 mutation. Epileptic Disord. 2020;22:659–63. 33063670 10.1684/epd.2020.1204

[24] Chen M, von Mikecz A. Proteasomal processing of nuclear autoantigens in systemic autoimmunity. Autoimmun Rev. 2005;4:117–22. 15823496 10.1016/j.autrev.2004.08.038

